# PRANAS: A New Platform for Retinal Analysis and Simulation

**DOI:** 10.3389/fninf.2017.00049

**Published:** 2017-09-01

**Authors:** Bruno Cessac, Pierre Kornprobst, Selim Kraria, Hassan Nasser, Daniela Pamplona, Geoffrey Portelli, Thierry Viéville

**Affiliations:** ^1^Biovision Team, Inria, Université Côte d'Azur Sophia Antipolis, France; ^2^Inria, Mnemosyne Project Team Bordeaux, France

**Keywords:** retina simulator, spike train statistics, population coding, maximum entropy, Gibbs distributions, large scale spiking activity, spike train generation, multi-electrode array recordings

## Abstract

The retina encodes visual scenes by trains of action potentials that are sent to the brain via the optic nerve. In this paper, we describe a new free access user-end software allowing to better understand this coding. It is called PRANAS (https://pranas.inria.fr), standing for Platform for Retinal ANalysis And Simulation. PRANAS targets neuroscientists and modelers by providing a unique set of retina-related tools. PRANAS integrates a retina simulator allowing large scale simulations while keeping a strong biological plausibility and a toolbox for the analysis of spike train population statistics. The statistical method (entropy maximization under constraints) takes into account both spatial and temporal correlations as constraints, allowing to analyze the effects of memory on statistics. PRANAS also integrates a tool computing and representing in 3D (time-space) receptive fields. All these tools are accessible through a friendly graphical user interface. The most CPU-costly of them have been implemented to run in parallel.

## 1. Introduction

The retina is one of the most developed sensing devices (Gollisch and Meister, 2010; Masland, 2011, 2012). It transforms the incoming light into a set of electrical impulses, called spikes, which are sent asynchronously to higher level structures in the visual cortex through the optic nerve. Although Cajal's neuron doctrine was postulated more than one century ago, how information is encoded and transmitted by neurons is still not entirely understood today. Especially, the role of spatio-temporal correlations in population coding raises up deep theoretical and practical questions that are far from being answered (Rieke et al., 1996; Cessac and Palacios, 2012), particularly for the visual information transmitted from the retina to the visual cortex. To address these questions and make progress in their understanding, one needs to develop joint modeling and experimental studies with efficient software to analyse data. In this paper we present a new Platform for Retinal ANalysis And Simulation called PRANAS (https://pranas.inria.fr). It was designed as a user-friendly tool dedicated to neuroscientist community in a large sense, i.e., not only experienced computational neuroscientists. It has two main goals, analyse retina data, especially spatio-temporal correlations, and simulate the spike response of the retina to a visual flow. This makes this tool a unique platform to better understand how the retina works.

The first goal of PRANAS is to provide methods to analyse retinal recordings at single cell and population levels. With the advent of new techniques, the recording of the simultaneous activity of groups of neurons provides a critical database to unravel the role of specific neural assemblies in spike coding. The acquisition capacity of Multi-Electrode Arrays (MEA) has been exponentially increasing over years (Stevenson and Kording, 2011). Systems like the 256–MEA has become a standard although new high-density MEA are now available such as the APS CMOS 4096–electrodes (Ferrea et al., 2012). Using these systems, one can record from hundreds to thousands of neurons simultaneously in the retina (and more generally *in vivo* or *in vitro*, from cultures of neurons, and from other brain areas). As one gains more intuitions and results on the importance of concerted activity in spike trains, models are developed to extract, from animal recordings, possible canonical principles underlying spike coding. This is a major challenge with many potential outcomes. Beyond the dream that population coding is ruled by a few first principles, several applications could emerge such as the development of artificial systems having similar levels of performance as biological systems. To this end, accurate tools are required to analyse and compare spike trains, experimental or computer generated ones. Progress has been made recently to analyse spike trains (Schneidman et al., 2006; Shlens et al., 2006; Marre et al., 2009; Roudi et al., 2009a,b; Tkačik et al., 2010; Roudi and Hertz, 2011; Tkačik et al., 2013). Especially, the spatio-temporal aspects (memory) and causality have been shown to be relevant for exploring neural activity (Cessac and Palacios, 2012). For instance, Vasquez et al. (2012) and Tang et al. (2008) demonstrated the importance of temporal statistics and Nasser et al. (2013b) and Nasser and Cessac (2014) developed new tools to analyse spatio-temporal activity for large scale spiking networks. These methods shed a new light on spike train analysis. However, they require time and expertise to be implemented efficiently, making them hard to use. The idea of developing a new software came from our motivation to share these recent developments with the neuroscience community in a broad sense. PRANAS integrates all our expertise in terms of spike trains statistical analysis. Note that other methods analyzing correlations in massively parallel data using completely different approaches have also been proposed (Hillar and Effenberger, 2015; Takahashi et al., 2015; Torre et al., 2016; Quaglio et al., 2017).

The second goal of PRANAS is to provide a customizable retina simulator which could evolve in synergy with experimental data analysis. Currently, there is a large and expanding body of literature concerning models of retinal processing. There are three main classes of models. The first class regroups the linear-nonlinear-poisson (LNP) models (Odermatt et al., 2012). LNP models can simulate the spiking activity of ganglion cells (and of cortical cells) in response to synthetic or natural images (Carandini et al., 2005) but they voluntarily ignore the neuronal mechanisms and the details of the inner retinal layers that transform the image into a continuous input to the ganglion cell (or any type of cell) stages. The second class of models has been developed to serve as a front-end for subsequent computer vision task. They provide bio-inspired modules for low level image processing. One interesting example is given by Benoit et al. (2010) and Hérault (2010), where the model includes parvocellular and magnocellular pathways using different non-separable spatio-temporal filter that are optimal for form- or motion-detection. The third class is based on detailed retinal models reproducing its circuitry, in order to predict the individual or collective responses measured at the ganglion cells level (Pelayo et al., 2004; Wohrer and Kornprobst, 2009; Lorach et al., 2012; Martinez-Alvarez et al., 2013). In PRANAS, we are interested in this third class of models because they allow to explore several aspects of retinal image processing such as (i) understanding how to reproduce accurately the statistics of the spiking activity at the population level (Nasser et al., 2013a), (ii) reconciling connectomics and simple computational rules for visual motion detection (Kim et al., 2014), and (iii) investigating how such canonical microcircuits can implement the different retinal processing modules cited in e.g., Gollisch and Meister (2010). More precisely, the PRANAS platform has integrated and extended the VIRTUAL RETINA simulator (Wohrer and Kornprobst, 2009)^1^ initially developed in our team to do large scale retina simulations. VIRTUAL RETINA has been used in several theoretical studies (Masquelier, 2012; Mohemmed et al., 2012; Basalyga et al., 2013; Doutsi et al., 2015a,b; Vance et al., 2015).

This paper, aiming at presenting this new platform PRANAS, is organized as follows. In Section 2, we give an overview of PRANAS and compare it with a selection of other tools currently available focusing on spike train analysis methods. In Section 3, we present the main features of the software. Illustrations and step by step procedures are given in several cases allowing readers to reproduce them. In Section 4, we discuss future developments.

## 2. General presentation

PRANAS targets a broad community of scientists interested in exploring spike coding, in particular at retina level. It provides new tools for analyzing spike trains at the population level and several methods to generate them, either with a prescribed statistics (including spatio-temporal correlations) or by emulating the retinal response to a visual scene. This is done through a user-friendly Graphical User Interface (GUI). PRANAS runs on multiple platforms (Linux, Mac OS, Windows) and it supports parallel architectures. The software is provided as binary code or as source code on request upon acceptance on the terms of the license given on the website (http://pranas.inria.fr). Documentation, tutorials, and samples of spike trains are also available.

**Figure 1:**
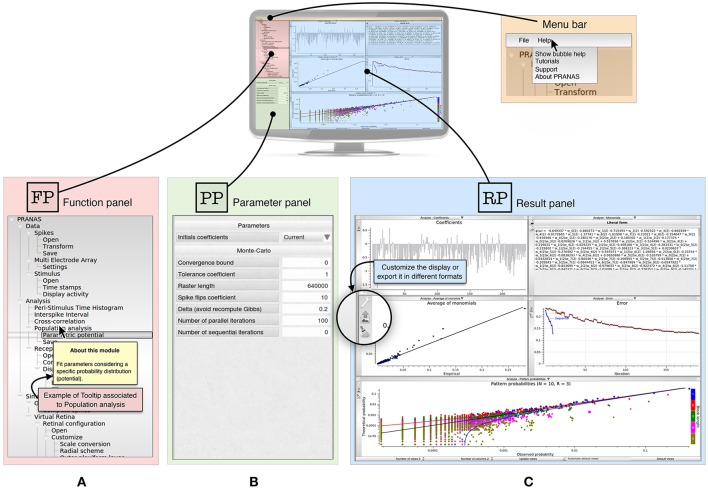
Presentation of PRANAS GUI panels. **(A)** Function panel (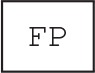) is the main panel containing Input/Output interfaces and functions. It is organized in three sections: Data, Analysis and Simulation. Note that in the Help option from the menu bar users can select Show bubble help which will trigger tooltips giving information for each function. **(B)** Depending on the function selected, the parameter panel (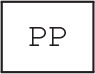) gives the opportunity to set the corresponding parameters. **(C)** Result panel (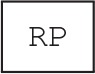) can show all results the user want. The number of sub-panels and the windows to show can be changed. Note that for each result shown in a sub-panel, the user can customize the display or export it in different formats.

The GUI of PRANAS (version 1.0.0) has three main panels, as shown in Figure 1:

- Function panel (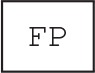) is the main panel containing Input/Output interfaces and functions. It is organized in three sections: Data, Analysis and Simulation. (see Section 3 for more details).
- The parameter panel (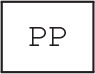) allows one to set parameters related to the chosen function from the function panel.
- The results panel (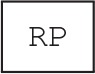) contains the results. Several results can be displayed simultaneously in different subpanels, and each result can be exported as a figure in a variety of formats (e.g., PDF, PNG, JPG). The user can change the Number of views and which result should be displayed in each subpanel. In most subpanels, there are icons on the left-hand side to open, save, export or change plot settings (see Figure 1C). Another example is shown in **Figure 4A** (Stimulus view) where the user can export, show/hide grid and spikes and select neurons graphically.

Finally, another convenient feature of the software is that user can save what he has done in term of analysis in a HDF5 file (Zordan et al., 2015) (File>Save) and load it later to continue the work.

PRANAS is implemented in C++. It has its own dedicated libraries. It also uses other libraries for storage (HDF5), analysis (GSL, SFMT), display and GUI (Qt4, Qwt, VTK, CImg). Some parts of the software, especially the statistical estimations, run on multiple processors thanks to OpenMP framework. The software takes all the available processors in the machine automatically, without any interaction with the user. Parallelization allows to boost heavy computations and save processing time and memory.

To our best knowledge there is no other platform integrating together the functionalities proposed by PRANAS, to both analyse and stimulate retinal activities. There are however several platforms performing efficient spike train analysis. Table 1 provides a qualitative comparison between PRANAS and a selection of such tools for spike train analysis. We note that the implementation of PRANAS as a stand-alone application in C++/Qt4 rather than a library (such as, e.g., the FIND toolbox) makes this tool readily available without the need of an external interpreter, such as Python or the commercially available Matlab suite (MathWorks, Natick, VA). Thus, access to PRANAS functions from a scripted analysis is performed via command line calls (see Section 4).

**Table 1:** Comparison between a selection of existing software for spike train analysis.

	**CX**	**FD**	**NT**	**SL**	**NY**	**OY**	**ET**	**SR**	**NE**	**SV**	**PS**
**Version Language**	**2.12 Matlab**	**2.0 Matlab**	**2.0 Matlab**	**1.00 Matlab**	**0.1 Python**	**0.3.5 Python**	**0.3.0 Python**	**0.3-2 R**	**5.037 VB**	**0.4.2 Python**	**1.0.0 C++**
GUI	•	•		•		•			•	•	•
Scripting	•	•	•	•	•	•	•	•	•		
Free binary	•	•	•	•	•	•	•	•	•	•	•
File formats (Nex)		•		•	•	•	•		•	•	•
File formats (HDF5)		•				•	•			•	•
Rate histogram PSTH				•	•		•		•	•	•
Population PSTH	•	•		•	•		•		•	•	•
ISI	•	•		•	•		•	•	•	•	•
Cross-correlograms	•	•		•	•		•	•	•	•	•
Joint spike and stimulus visu.											•
STA					•	•	•		•		•
PCA									•		
Spectral analysis	•				•	•	•		•	•	
MaxtEnt (x/x-t)			GLM								•
Raster generation				•	•						•
…with Poisson/Non Poisson					•	•					•
…with VIRTUAL RETINA											•

*Abbreviations for software names (first and last initials) have been chosen for the presentation. Following software is discussed: CX, CHRONUX (Ince et al., 2010); FD, FIND (Meier et al., 2008); NT, NSTAT (Goldberg et al., 2009); SL, SIGTOOL (Lidierth, 2009); NY, NEUROPY (Spacek et al., 2008); OY, OPENELECTROPHY (Garcia and Fourcaud-Trocmé, 2009); ET, ELEPHANT; SR, STAR (Pouzat and Chaffiol, 2009); NE, NEUROEXPLORER; SV, SPYKEVIEWER (Pröpper and Obermayer, 2013); PS, PRANAS. Note that features selected in this table have essentially been chosen according to what PRANAS does. It is not an exhaustive list and information applies to the time of writing. Software we mention can have additional features not commented herein*.

## 3. PRANAS main functions

### 3.1. Data

In section Data, user can load spike trains files. Spikes may come either from real cells recordings or from a simulated spiking neural network. They can be imported from different formats such as simple text file (.txt), DAT file for Windows (.dat), NEXUS file (.nex) or HDF5 file format (.hdf5, see Zordan et al., 2015) allowing to store not only the spikes but also other information related to experimental protocole (e.g., time stamps, MEA characteristics).

Depending on how the spikes were generated, the user can also load other information such as:

- The MEA configuration for animal cell recordings, allowing the spiking activity to be displayed relative to the grid of electrode.
- The sequence of images in case of a visual experiment, allowing the spiking activity to be displayed together with the stimulus but also to emulate a retinal response (see Section 3.3.2).
- The image time stamps, i.e., the precise time at which each image was shown, allowing to perform Spike-Triggered-Average (STA) (Chichilnisky, 2001).

### 3.2. Analysis

#### 3.2.1. Classical analysis

**Figure 2:**
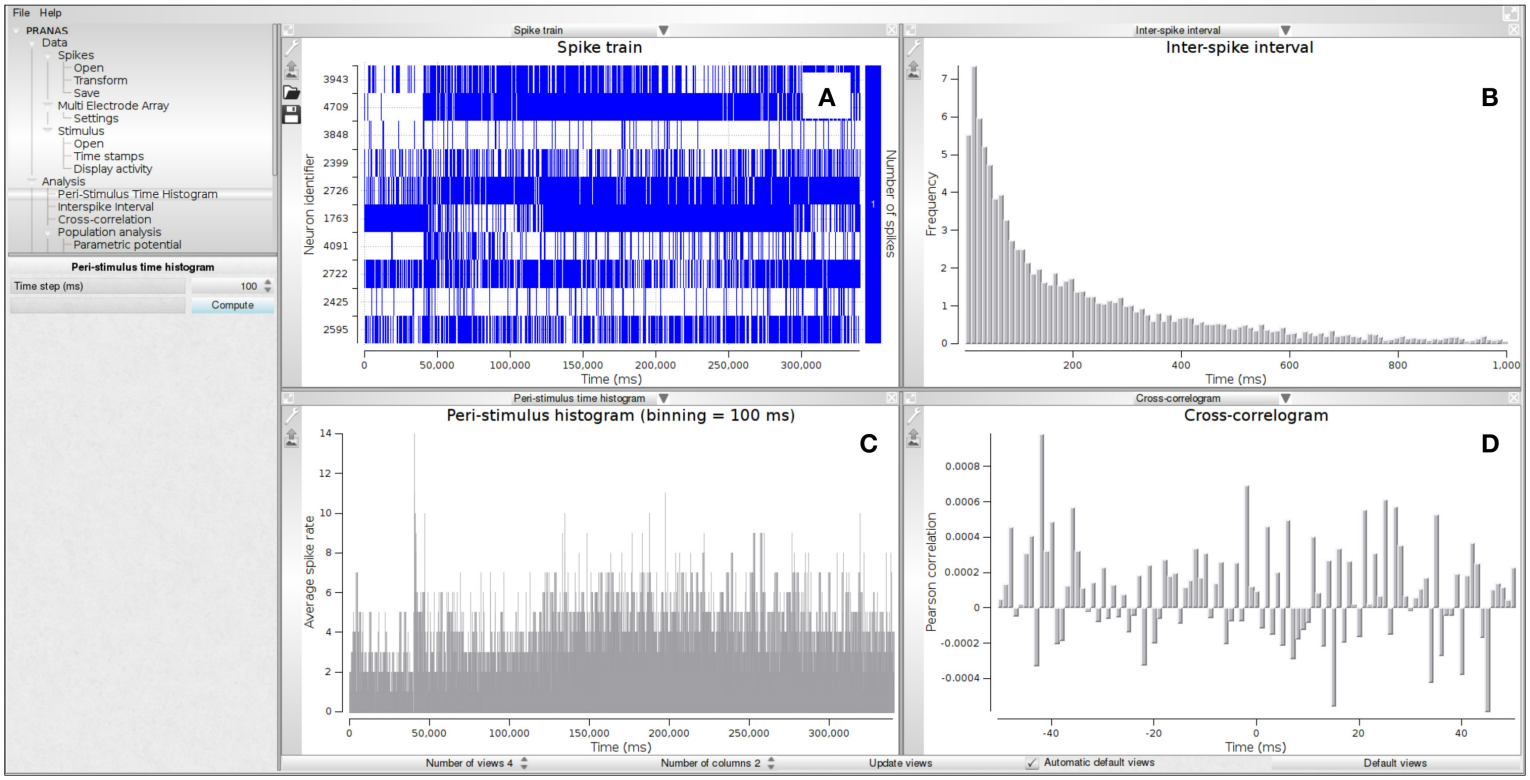
Example of classical analysis for a spike train shown in sub-panel **(A)**. The following estimations are shown: **(B)** Inter-spike interval (ISI), **(C)** Peri-stimulus time histogram (PSTH) and **(D)** cross-correlogram. Data from electrode array recording of a mouse retina in response to a moving pattern.

Once the spikes are available (simulated or imported), different analysis can be performed starting with basic visualizations and classical analysis such as peri-stimulus histogram, interspike interval, and cross-correlation (see Figure 2 and Example 1). The number of neurons used for the analysis can be of several thousands, although the user can select subsets of neurons according to different criteria, e.g., individual activity or receptive field localization (if applicable, i.e., if data allows to compute receptive fields). Even for a large population of neurons (about 4,000 neurons), on any modern computer, the computational time of most of the functions in the classical analysis is neglectable.

**Example 1** How to obtain the peri-stimulus time histogram (PSTH) on a selection of neurons having the highest spike rates?

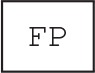
 Data>Spikes>Open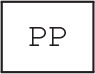 Browse and select the file of spikes.
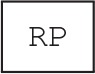
(Optional) In the sub-panel showing the list of neurons, click on Spike rate to sort neurons with respect to their averaged spike rate, select the set of neurons with higher spike rate and click on Keep selected.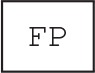 Analysis>Peri-stimulus time histogram.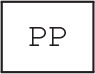 Choose the bin size (time step) in milliseconds and click on Compute.

#### 3.2.2. Population analysis

With the current evolution of Multi-Electrode Array (MEA) recordings devices, it is possible to record simultaneously thousands of neurons (Ferrea et al., 2012). This opens up the possibility of analyzing the collective neuronal population activity, namely its spatio-temporal correlations. Characterizing spatio-temporal statistics is a fundamental step toward extracting general population coding principles in neuronal networks. For example, it has been observed in several vertebrate species that, even if the pairwise correlations of retinal ganglion cell are weak, they are necessary to explain the statistics of spikes (Ginsburg et al., 1984; Schneidman et al., 2006; Shlens et al., 2006; Tkačik et al., 2010; Greschner et al., 2011).

Analyzing spatio-temporal correlations is, however, a notoriously hard problem from a statistical perspective. The point is not only to measure pairwise or higher order correlations but also to address adequately the question of their significance in neuronal coding taking into account constraints such as limited statistical sample, the reliability of statistical tests, the hypotheses underlying mathematical models and risks of overfitting (Roudi et al., 2009a,b). One possible way of solving this problem is to use Gibbs distributions. Initially introduced in statistical physics by Boltzmann and Gibbs (Jaynes, 1957) but currently used in a broader context, this general class of probability distributions, constitutes a canonical paradigm to explain the spike statistics reproducing as close as possible empirical correlations, without adding additional unnecessary assumptions (Schneidman et al., 2006; Shlens et al., 2006; Marre et al., 2009; Tkačik et al., 2010, 2013; Ganmor et al., 2011a,b). This method has been used to study pairwise correlations (see Vasquez et al., 2012; Nasser et al., 2013b; Nasser and Cessac, 2014) as well as more general spatio-temporal correlations (Cessac et al., 2009; Vasquez et al., 2010; Cofré and Cessac, 2013; Nasser et al., 2013b; Cofre and Cessac, 2014; Herzog et al., 2014; Ravello et al., 2015; Herzog et al., in preparation).

PRANAS provides a toolbox to compute a Gibbs distribution from the neuronal data i.e., to estimate a probability distribution, reproducing as close as possible empirical space- and time-statistics of a raster, without adding additional unnecessary assumptions (maximizing entropy)^2^. In the current version, the population analysis toolbox of PRANAS focuses on stationary distributions, i.e., the statistics are invariant under time-translation. We give first a brief explanation of Gibbs distribution before explaining how to use PRANAS (see Vasquez et al., 2010; Nasser et al., 2013b; Nasser and Cessac, 2014 for more details).

Below, rasters are denoted by ω. We assume that time has been discretized with some time bin. The state of neuron *i* at time *t* is denoted by ω_*i*_(*t*)∈{0, 1}. We note by ω(*t*) the vector ω_*i*_(*t*) (it tells us the spiking state of neurons at time *t*). Therefore, a raster ω is mathematically a matrix with *N* lines (number of neurons) and *T* columns (time bins in the raster). Obviously, it is not stored this way in memory (we do not store 0 s). A Gibbs distribution is a probability measure μ defined by a function *E* (also called “energy”). The probability of observing a raster ω of time length *T*, μ[ω] is proportional to *e*^*E*(ω)^ where $E\left(\omega \right)=\overset{T}{\underset{t=1}{\sum }}\phi \left(\omega \left(t\right)\right)$. In the following ϕ is called “potential.” A paradigmatic example of Gibbs distribution appears in the Ising^3^ model. Here ϕ(ω(*t*)) = ∑_*i*_*b*_*i*_ω_*i*_(*t*)+∑_*i,j*_*J*_*ij*_ω_*i*_(*t*)ω_*j*_(*t*) where the sums hold on neurons indices. The terms *b*_*i*_, *J*_*ij*_ are parameters tuning the probability whereas the terms ω_*i*_(*t*), ω_*i*_(*t*)ω_*j*_(*t*) depend on the spike configuration and are called “interactions” (self-interaction for the term ω_*i*_(*t*), and pairwise interactions ω_*i*_(*t*)ω_*j*_(*t*)). Here parameters *b*_*i*_, *J*_*ij*_ are independent on time (stationarity assumption) so it is sufficient to define the potential ϕ at time *t* = 0.

In the Ising model, there is no coupling of spikes at different times so that the probability of observing a raster of length *T* factorizes into probabilities of spike states ω(*t*): consecutive time events are therefore independent. We say the statistical model has “no memory” in contrast to the Markovian model presented now. A natural generalization of the Ising form is indeed to define the potential ϕ as:

(1) $$\begin{array}{r} \phi \left(\omega \right)=\overset{L}{\underset{l=0}{\sum }}h_{l}m_{l}\left(\omega \right) \end{array}$$

The terms *h*_*l*_ are parameters tuning the probability. They correspond to *b*_*i*_, *J*_*ij*_ in Ising model but they tune more general spike interactions. Again, these parameters are independent of time (stationarity) so that we can define the potential ϕ starting from an initial time 0. The main difference with Ising model is that now interactions involve spikes at different times *t*_1_, …, *t*_*n*_. These interactions correspond to the terms *m*_*l*_(ω), with the general form ω_*i*_1__(*t*_1_)…ω_*i*_*n*__(*t*_*n*_), i.e., it involves spike events occurring at different times. As an immediate consequence, consecutive time events are not independent anymore. One can show the Gibbs distribution is, in this case, the invariant probability of a Markov chain. Thus, statistics involves memory and has non-vanishing time correlations. For historical reasons, related to the development of our research work, we call from now the *m*_*l*_s “monomials” instead of “interactions.” The monomials correspond thus to the conjunction of events in the raster, varying the space and time. For instance, the condition “neuron zero” is firing at time one, neuron two is firing at time zero and neuron three is firing at time two” corresponds to *m*(ω) = ω_0_(1)ω_2_(0)ω_3_(2). More generally, the times *t*_*k*_ defining a monomial are chosen in the interval [0, *D*] where *D* is a positive integer characterizing the memory depth of the Markov chain associated with the Gibbs distribution. The model range is given by *R* = *D*+1. The potential range is directly connected to the complexity of the algorithm analyzing data, as the higher the range the higher the computational time and memory load. Gibbs distributions satisfy a variational principle: maximizing the statistical entropy under the constraints that the average value of each monomial, μ[*m*_*l*_] has a fixed value. Gibbs distributions are thus also called Maximum Entropy (MaxEnt) distributions. In our case, this value is equal to average empirical value π[*m*_*l*_] computed from an experimental raster. In other words, here is what our algorithm does: given an initial form of the potential ϕ, fixed by the user (see below), one seeks the parameters *h*_*l*_ maximizing the statistical entropy under the constraints μ[*m*_*l*_] = π[*m*_*l*_]. In general, it is not possible to have the strict equality μ[*m*_*l*_] = π[*m*_*l*_] so one tries to approach it at best. Equivalently, we minimize the Kullback-Leibler divergence between μ, the “model” and π the empirical measure (“data”). In the ideal case of a raster infinite in time, this is a convex problem, and therefore there is a unique solution. For the realistic case, the input raster is finite, and several solutions can solve the problem (Cessac and Palacios, 2012). The algorithm starts from an initial guess of the *h*_*l*_s and computes the corresponding averages μ(*m*_*l*_) using a Monte Carlo method. From this, it computes a variation of the *h*_*l*_s giving, if possible, a lower Kullback-Leibler divergence (see Nasser and Cessac, 2014 for details). We proceed this way until no improvement in the Kullback-Leibler divergence is observed any more. At this point, modifying some parameters (see next paragraph) can nevertheless still improve the minimization.

Let us now describe how to compute Gibbs distributions with PRANAS. There are many (an exponential number) a priori possible potentials. In PRANAS we propose four predefined potentials although the user can also define his own one. The options are the following. Here we characterize the potential by the list of monomials M which compose it.

- Bernoulli model: It takes into account individual neurons activity where all neurons are independent. The potential reads $\phi \left(\omega \right)=\overset{N}{\underset{l=1}{\sum }}h_{l}\omega_{l}\left(0\right)$ where *N* is the number of neurons. Hence, monomials are of type “neuron *i* is firing at time *t*” (Example: $M=\left\{\omega_{1}\left(0\right),\omega_{2}\left(0\right),\omega_{3}\left(0\right)\right\}$ for 3 neurons). This model has range 1.
- Ising model: This paradigmatic model from statistical physics has been used in neuroscience in several papers such as Schneidman et al. (2006), Shlens et al. (2006), Tkačik et al. (2006), and Tkačik et al. (2009). The corresponding potential has been introduced above. The monomials are of type “neuron *i* is firing at time *t*” and “neuron *i* and neuron *j* are simultaneously firing at time *t*.” Thus events are instantaneous, and this model does not involve memory and causality (consecutive times are independent under μ, the Gibbs distribution). This model has range 1 (Example for 3 neurons: $M=\left\{\omega_{1}\left(0\right),\omega_{2}\left(0\right),\omega_{3}\left(0\right),\omega_{1}\left(0\right)\omega_{2}\left(0\right),\omega_{1}\left(0\right)\omega_{3}\left(0\right),\omega_{2}\left(0\right)\omega_{3}\left(0\right)\right\}$).
- Pairwise + Triplets model: This model has been introduced by Ganmor et al. (2011a,b). In addition to Ising terms, there are triplets of interactions (e.g., ω_1_(0)ω_2_(0)ω_3_(0)). This model too has range 1.
- Pairwise model: This is an extension of Ising model where monomials are of type “neuron *i* is firing at time *t*” (single events) and “neuron *i* is firing at time *t* and neuron *j* is firing at time *t*+*k*,” 0 ≤ *k* ≤ *D* (pairwise events). This model integrates memory and causality via time dependent pairwise interactions. (Example with range 2 and 2 neurons: $M=\left\{\omega_{1}\left(0\right),\omega_{2}\left(0\right),\omega_{1}\left(0\right)\omega_{2}\left(0\right),\omega_{1}\left(0\right)\omega_{1}\left(1\right),\omega_{2}\left(0\right)\omega_{2}\left(1\right),\omega_{1}\left(0\right)\omega_{2}\left(1\right),\omega_{1}\left(1\right)\omega_{2}\left(0\right)\right\}$).
- User: Here the user defines his own potential (Example $M=\left\{\omega_{1}\left(0\right),\omega_{2}\left(0\right),\omega_{3}\left(0\right)\omega_{1}\left(0\right)\omega_{2}\left(1\right)\omega_{3}\left(2\right),\ldots \;\right\}$).

**Example 2** How to calculate the Gibbs distribution of a spike train?

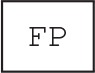
 Data>Spikes>Open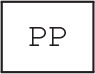 Browse and select the file of spikes.
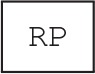
(Optional) In the sub-panel showing the list of neurons, select a subset of neurons and click on Keep selected.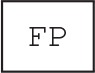 Analysis>Population analysis.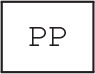 Define the Potential, either from a file or by setting the type (e.g., pairwise) and the range^4^ (e.g., *R* = 3). Set the Maximal pattern length. For example, if you select 2, the program will seek *in the data* all spike events occurring within 2 successive time steps, and compare the probability of these events, predicted by the model, to the empirical probabilities. In Simulation, choose what you want to compute. Possible choices are:
  - Potential: Fits the coefficients of the potential.
  - Kullback-Leibler divergence: An estimation of Kullback-Leibler divergence derived from the entropy estimation in Strong et al. (1998) and the classical relations between K-L divergence, μ average of ϕ and entropy (Cessac and Palacios, 2012).
  - Pattern probabilities: The confidence plot.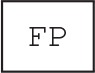 Analysis>Population analysis>Parametric potential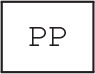 Choose Initial Coefficients: possible choices are Current (in the case when you have made a run before and you want to keep *h*_*l*_s) or 0. For the first run you can choose both as the initial parameters are anyway set to 0 (this corresponds to start from a Bernoulli models where spikes are independent with probability of occurrence $\frac{1}{2}$). Set parameters of the Monte Carlo method to speed up the process (see text for the role of each parameter).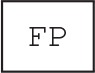 Population analysis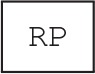 Compute to do the estimation. The red bar at the bottom indicates that computation is in progress.

Note that in PRANAS the user can fine-tune the probability estimation and speed up the computation in the first steps of the Monte Carlo method by acting on the following parameters (in 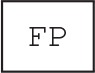 Analysis>Population analysis>Parametric potential):

- Convergence bound: The lower bound of error you require. Simulations stops when the code reaches this value.
- Tolerance Coefficient: Allows to filter monomials to compute *h*_*l*_s. For example, if the event *m*_*l*_ appears only, say 3 times in the raster you may consider that it is not significant and must be eliminated from the potential (1). In this case you set Tolerance Coefficient to 3.
- Raster length: The length of the Monte Carlo raster used to compute the average of monomials for the current values of *h*_*l*_s. This number must increase as you get closer to the solution (typically when you do not get any improvement in the error).
- Spike flips coefficients: This defines the number of flips per neuron and per iteration in Monte Carlo methods (Nasser and Cessac, 2014).
- Delta: If the distance between predicted and empirical distribution is smaller than Delta the Monte Carlo raster is not recomputed. Instead, the average value of monomials is computed via linear response (Nasser and Cessac, 2014). The number must be decreased as you get closer to the solution (typically when you do not get any improvement in the error).
- Number of parallel iterations: We use two kinds of updating, based upon Dudík et al. (2004): parallel or sequential. Parallel update sets all *h*_*l*_s in one step. Number of parallel iterations tells how many parallel updating of *h*_*l*_s are done before the program stops (and e.g., plots the confidence plot, when selected).
- Number of sequential iterations: Sequential update. It computes only one *h*_*l*_ at each step. It is better to use it at the end of the computation, to fine-tune coefficients.

**Figure 3:**
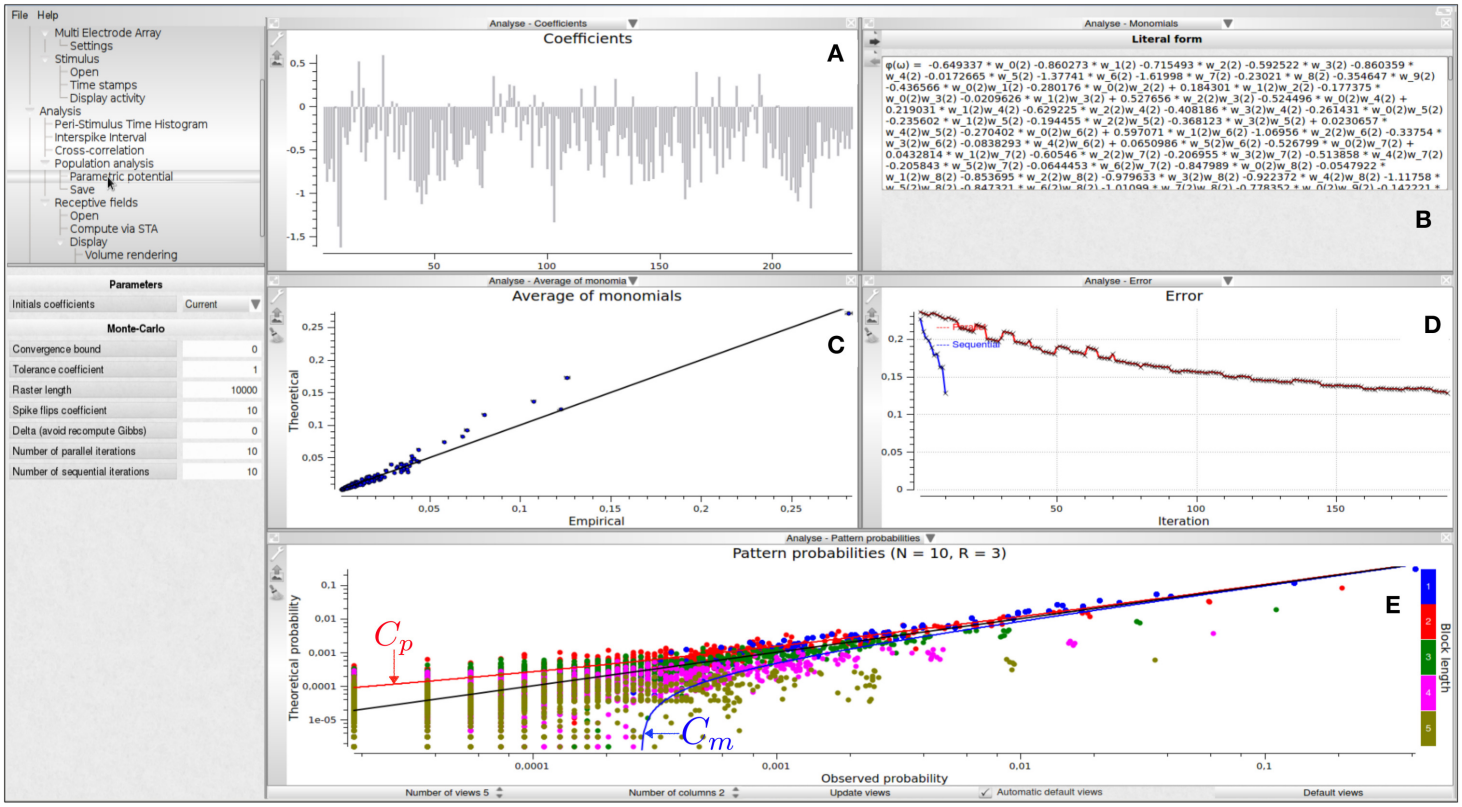
Population analysis view with *N* = 10, *R* = 2. **(A)** Graphic view of the potential's coefficients values; the abscissa corresponds to an index associated to a prescribed order on monomials: the *N* first coefficients corresponds to single events (hence are related to firing rate); by clicking on the histogram bar one makes appear the coefficient value and the corresponding monomial. Ordinate give the value of the coefficient attached to the corresponding monomial. **(B)** Literal form of the potential. **(C)** Theoretical average value of monomials vs. their empirical average value. To which extents the model predicts the empirical data is drawn with this representation. This allows checking that constraints on empirical averages are respected by the model. **(D)** Decay of estimation errors vs. the number of iterations allowing us to graphically check the convergence of the estimation algorithm (red curve). In the figure several successive runs have been performed. The blue curve shows the evolution of error after each of these trials. **(E)** Confidence plot. *C*_*p*_, *C*_*m*_ corresponds, respectively, to the upper and lower 3σ bound associated with Central Limit Theorem (see text).

Figure 3 shows different visualizations of the results. In Figure 3A, we show a graphical view of the potential's coefficients. In Figure 3B, we show the literal form of the potential ϕ(ω). In this example, it is

(2) $$\begin{array}{l} \phi \left(\omega \right)=-0.860273\;w_{1}\left(2\right)-0.715493\;w_{2}\left(2\right)+\ldots \\ -0.280176\;w_{0}\left(2\right)w_{2}\left(2\right)\\ +\cdots -0.120281\;w_{0}\left(2\right)w_{3}\left(2\right)w_{5}\left(3\right)+\ldots , \end{array}$$

where, e.g., *w*_0_(2)*w*_2_(2) corresponds to the event neuron zero and neuron two are firing at time two whereas −0.280176 is the corresponding coefficient *h*_*l*_ of this term in the potential. In Figure 3C, we show a plot of monomials average value, with empirical average on the abscissa and theoretical average (predicted by μ) on the ordinate. In Figure 3D, we show the evolution of the Hellinger^5^ distance between the model predictions and empirical data, vs. the number of iterations. Hellinger distance is natural here as it relies directly on the estimation of the average value of monomials. It provides a quantitative measure of the goodness of fit. Finally, in Figure 3E we show a *confidence plot*. This figure consists of plotting, in log scale, on the abscissa the empirical probability of observed spike blocks and on the ordinate the expected probability of those blocks for the model μ. If the matching were perfect one would have all points aligning on the diagonal. This perfect matching is only possible, however, if the input spike train were infinite. For finite spike trains fluctuations about the exact probability are observed, ruled by Central Limit Theorem. Namely, fluctuations are Gaussian with a variance σ_*l*_, depending on the monomial *l* and proportional to $\frac{1}{\sqrt{T}}$, *T* being the raster length (number of time bins). Around the diagonal are drawn error bounds corresponding to 3σ_*l*_, where σ is an estimated empirically. These bounds define a confidence region: if the Gibbs distribution is a perfect estimation of the input raster then points in the confidence plot are distributed inside the confidence region with a probability of 99.7% of the expected averages. The confidence plot provides a qualitative measure of the goodness of the fit.

Reducing the computation time has been a primary concern for us during the development phase. In fact, the most time-consuming routine of PRANAS is for inferring the Gibbs Potential. To face this problem, we introduced a new algorithm based on Monte Carlo sampling (Nasser et al., 2013b). For a large number of neurons, the Monte Carlo-based algorithm offers better computation time than the algorithm proposed in Vasquez et al. (2010). With a cluster of 64 cores of 2.4 GHz speed, PRANAS needs the following amount of time to compute a target distribution (1 iteration): 5 min for 20 neurons with a pairwise model *R* = 2, 10 min for 40 neurons with an Ising model. To infer the Gibbs Distribution, one needs 100–200 iterations on the *h*_*l*_ estimation. For more details on the scalability of this method see Nasser et al. (2013b).

#### 3.2.3. Receptive fields estimation

Beyond tools to analyse spike trains at the single cell and population levels, PRANAS provides specific instruments in the case when spike trains come from evoked activity of neurons from the retina. The user can upload MEA characteristics, the sequences of images of the stimulus and the time stamps. Given this information, PRANAS provides a method to estimate the neuron's receptive fields. The method available in version 1.0.0 is Spike Triggered Average (STA) (Chichilnisky, 2001). The STA can be computed for a single neuron, a subset of neurons or the entire population. Example 3 describes the general procedure.

**Example 3** How to estimate receptive fields with STA method?^6^

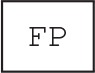
 Data>Spikes>Open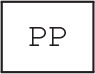 Browse and select the file of spikes. Note that the user can select a subset of neurons as in Example 1.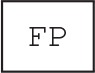 Data>Multi-Electrode Array>Settings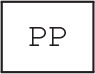 Define the MEA configuration, so that, if neurons have a position relative to the array, the spike activity can be displayed at the correct location.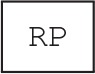 Stimulus view shows the activity of individual neurons w.r.t. MEA array. The user may choose a region of interest to select a subset of neurons. Note that if you have a TEXT file for spikes, no position is available, and we chose to arrange neurons following their order, by rows and columns.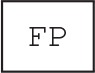 Data>Stimulus>Open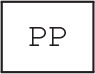 Browse to choose the directory containing the sequence of white noise images. Note that all images should be in this directory and ordered by name. All standard formats are accepted (e.g., PNG, JPG, TIFF).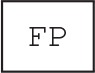 Data>Stimulus>Time stamps: Time stamps of the exact offset of each image during the experiment.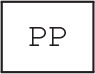 Browse and select the file. Time stamps can be imported from a .txt (row *n* contains the starting time of image *n*) or HDF5 (Zordan et al., 2015).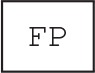 Analysis>Receptive fields>Compute viaSTA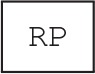 Output directory: Choose the directory where the receptive fields will be saved, as a sequence of PNG files and in HDF5 format. Choose the number of images to be averaged before each spike with Time depth (number of slices). Then click on Compute.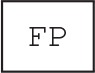 Analysis>Receptive fields>Display allows four types of visualization.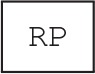 The user can display the receptive field of interest by selecting the neuron in Receptive Fields subpanel and Update views.

**Figure 4:**
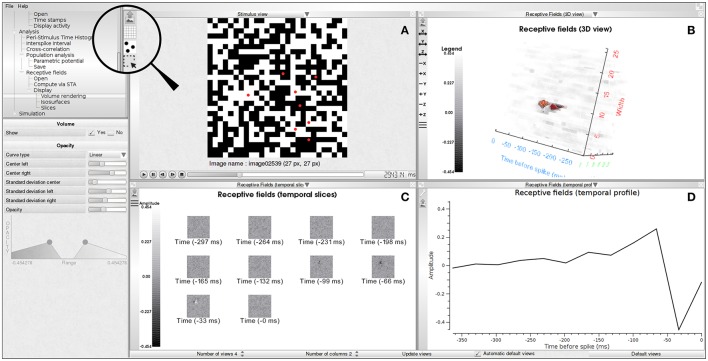
Receptive field estimation using the STA approach. **(A)** White noise image sequence with spiking activity superimposed as red dots. **(B)** 3D volume visualization of the receptive field of one neuron. **(C)** Temporal slices of the receptive field (*RF*(·, ·, *z*)). Slices are in increasing time order from top to bottom and left to right. Time period between two slices is 33 ms. **(D)** Temporal profile passing through the peak activity location of the spatio-temporal volume (*RF*(*x*_*M*_, *y*_*M*_, ·) where (*x*_*M*_, *y*_*M*_, *z*_*M*_) = argmax_*x, y, z*_*RF*(*x, y, z*)).

A receptive field is stored in a regular grid in a three-dimensional space. Each voxel in that grid, denoted by *RF*(*x, y, z*), stores the value of the receptive field at each spatial position (*x* × *s*_*d*_, *y* × *s*_*d*_) and temporal depth *z* × *t*_*d*_, where *s*_*d*_ and *t*_*d*_ denote, respectively, the spatial and temporal resolutions. The temporal resolution is estimated from the time stamps file as the average difference between two successive time stamps. The number of time slices of the receptive field, *n*_*f*_, is a user-defined parameter. The first spatial slice (*x, y, z* = 0) corresponds to the average spike triggered stimuli at time −(*n*_*f*_ − 1)*td* before the spike, the second slice (*x, y, z* = 1) corresponds to the average spike triggered stimuli at time −(*n*_*f*_ − 2) × *t*_*d*_ before the spikes and so on. In PRANAS we propose several displays of that volume (2D and 3D), as illustrated in Figure 4.

The time spent on the estimation of receptive fields depends on the parameters. For example, on a computer equipped with a Intel Core i7@2.8 GHz and 32 GB of memory it takes about 2 min to estimate the receptive fields of 4,000 neurons with 64 × 64 × 12 voxels each.

### 3.3. Simulation of spike trains

#### 3.3.1. Simulation of spike trains from statistics

PRANAS gives the possibility to generate spike trains from Gibbs distributions. This can be useful if, for example, one wants to generate spike trains with prescribed spatio-temporal correlations so as to test a statistical method of analysis. There are two different ways:

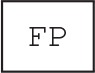
 Simulation>ComputeSpikes>GibbsRaster>current: if distribution comes from a population analysis (see Section 3.2.2).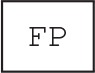 Simulation>ComputeSpikes>GibbsRaster>File: if distribution has been defined by the user and stored in a file containing the Gibbs potential form.

A Gibbs probability distribution (defined by Gibbs potential of the form 1) is naturally associated with a Markov chain, with memory *D* = *R*−1, whose transition probabilities can be computed from the potential ϕ (Cessac and Palacios, 2012; Vasquez et al., 2012; Cofre and Cessac, 2014). The invariant distribution μ of this chain is the Gibbs distribution associated to ϕ. Therefore, given a potential of the form (1) it is easy to generate a sample raster distributed according to μ using Monte Carlo method (Nasser et al., 2013b). PRANAS affords this functionality allowing to generate rasters with spatio-temporal correlations tuned by the parameters *h*_*l*_ in ϕ.

#### 3.3.2. Simulation of spike trains from a retina simulator

Another strength of PRANAS is to provide a way to generate spike trains that mimic retina's outputs. To do so, PRANAS has integrated and extended the VIRTUAL RETINA simulator (Wohrer and Kornprobst, 2009) formerly developed in our team^7^.

In a nutshell, VIRTUAL RETINA is a software to perform large-scale simulations of biologically-plausible retinas. Given a retina configuration which can be fully customized and an input video as visual stimulus, VIRTUAL RETINA simulates the spiking output for different cell types. VIRTUAL RETINA has been shown to reproduce a broad range of experimental data at single cell level, from salamander, cat and primate retinas, and has been used in several theoretical studies (Masmoudi et al., 2010; Masquelier, 2012; Mohemmed et al., 2012; Basalyga et al., 2013; Doutsi et al., 2015a,b; Vance et al., 2015). The underlying model includes a non-separable spatio-temporal linear model of filtering in the Outer Plexiform Layer, a shunting feedback at the level of bipolar cells, and a spike generation process using a network generalization of the noisy leaky integrate-and-fire neurons to model Ganglion Cells (GCells). Note that VIRTUAL RETINA was designed to be used via a command line (there was no GUI available).

By integrating VIRTUAL RETINA software into PRANAS platform, we allowed this simulation software to be used through a GUI. We also extended VIRTUAL RETINA with the aim to reproduce statistically coherent responses at a population level. Indeed, outputs of the initial VIRTUAL RETINA version were successfully compared to experimental data at single cell level but could not reproduce collective statistics at a population level. This is because GCells in the early version were not connected. They were modeled by independent leaky-integrate and fire neurons receiving their input from bipolar cells, but with no lateral connectivity (due to amacrine cells—ACells—in the retina). As a consequence correlations in GCells spikes were only due to statistics of the stimulus and overlapping receptive fields.

In the new version of the retina simulator embedded in PRANAS, GCells are connected laterally so as to explore the effects of connectivity on retinal responses to stimuli. More precisely, the IPL is now composed of discrete time leaky-integrate and fire neurons introduced in Soula et al. (2006) and studied mathematically in Cessac (2008, 2011). We have chosen this model because of its simplicity, fast response, and the fact that its collective dynamics are well known. In a nutshell, the model comes from the time discretization of the leaky-integrate and fire model whose sub-threshold dynamics reads:

(3) $$\begin{array}{r} C\frac{dV}{dt}=-g_{L}\left(V-V_{L}\right)+I\left(t\right)+\sigma_{B}B\left(t\right), \end{array}$$

for *V* < θ, where θ is the firing threshold. Here *C* is the membrane capacity, *g*_*L*_ the leak conductance, *V*_*L*_ is the leak reversal potential, *B*(*t*) is a Gaussian noise (mean zero and variance 1) and σ_*B*_ controls the amplitude of this noise. Finally, *I*(*t*) is an external current. In our case, this is the bipolar current coming from the OPL (see Wohrer and Kornprobst, 2009 for details).

Let $\tau_{L}=\frac{C}{g_{L}}$ be the leak characteristic time. We consider now a time discretization with a time step *dt* (here it is fixed, *dt* = 1 ms) and we set $\gamma =\left(1-\frac{dt}{\tau_{L}}\right)$. Note that *dt* has to be quite smaller than τ_*L*_ to have a reasonable description of the biophysics. Therefore γ ∈ [0, 1]. Then, the full dynamics (below and above threshold) of the membrane potential *V*_*i*_ of neuron *i* is given by:

$$V_{i}\left(t+1\right)=\gamma V_{i}\left(t\right)\left(1-Z_{i}\right)+\frac{I_{i}\left(t\right)}{C}+\frac{\sigma_{B}}{C}B\left(t\right),$$

where Z is the binary spike label:

$$Z_{i}=\left\{ \begin{array}{cc} 1,\; & \text{if }V_{i}\geq \theta ;\\ 0,\; & \text{otherwise.} \end{array}\right.$$

Thus *Z*_*i*_ is 1 whenever neuron *i* spikes. Here 1−*Z*_*i*_ models the reset. In this equation, neurons are not coupled. We add then a connectivity through synaptic weights *W*_*ij*_ where neuron *j* (pre synaptic) acts on neuron *i* (post synaptic) upon spiking. We have now:

(4) $$\begin{array}{r} V_{i}\left(t+1\right)=\gamma V_{i}\left(t\right)\left(1-Z_{i}\right)+\frac{I_{i}\left(t\right)}{C}+\frac{1}{C}\overset{N}{\underset{j=1}{\sum }}W_{ij}Z_{j}+\frac{\sigma_{B}}{C}B\left(t\right). \end{array}$$

For a complete description see Cessac (2008, 2011). Clearly, this modeling of lateral connectivity, although usual in the field of neural networks models, is rough when compared to the real lateral connectivity in the IPL involving amacrine cells having a complex dynamics. Elaborating more realistic coupling is under current investigations.

The simulator allows to tune the parameters leak (γ∈[0, 1]); neuronal noise (σ_*B*_ > 0); membrane capacity (*C*); threshold (θ) and the connectivity matrix (*W*). The connectivity between neurons is set through the connectivity matrix. One can simulate independent neurons by choosing none or connected neurons using sparse or dense schemes. One can also upload a predefined connectivity matrix offering the possibility to explore its impact on spike statistics. For example, we provide on the website an example of ACells-like connectivity pattern.

**Figure 5:**
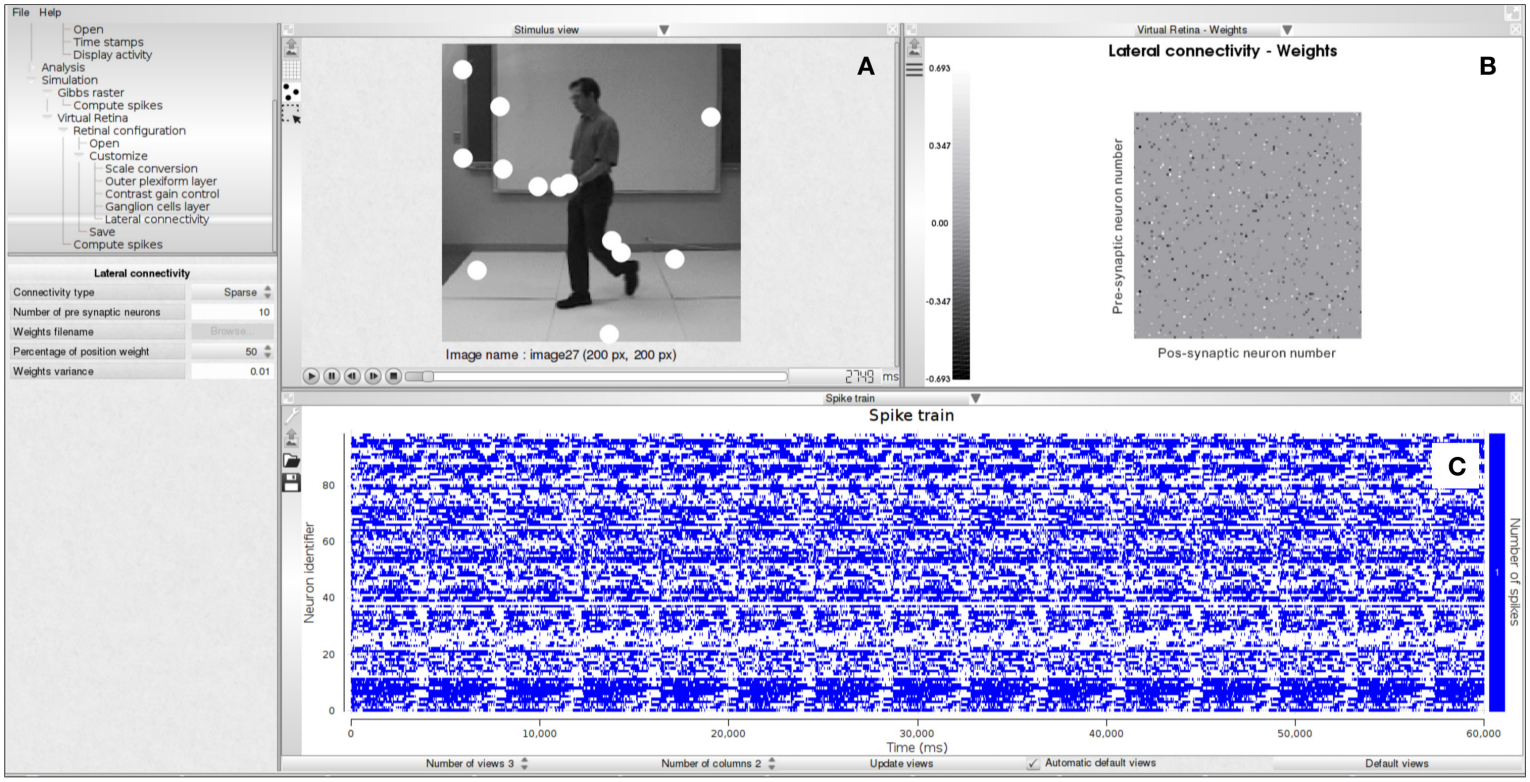
Spike generation from our retina simulator. **(A)** Input image sequence with spiking activity super-imposed (white blobs). **(B)** Connectivity matrix chosen for the simulation, here random. **(C)** Simulated spike train.

Example 4 shows how to generate spikes using PRANAS and Figure 5 illustrates an example of simulation. Note that user can find online retina configuration files and videos sequences to test the simulator.

**Example 4** Running the retina simulator^8^.

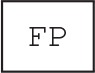
 Data>Stimulus>Open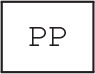 Browse to choose the directory containing the sequence.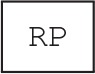 Stimulus sequences subpanel: The sequence of images is loaded with a default display duration of 100 ms per image (Display time column). This value can be manually set or defined from time stamps file (see Example 3).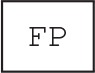 Simulation>Virtual Retina>Retinal configuration>Open^9^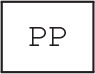 Browse and select the XML files containing the parameters of the retina.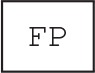 Simulation>Virtual Retina>Retinal configuration>Customize: Once loaded, the user can still change parameters of the retina.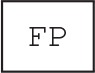 Simulation>Virtual Retina>Compute spike.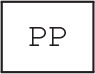 Compute to get the spikes.

The time spent in the generation of spikes with the retina simulator depends on the parameters. For example it takes about 6 min to generate the spikes corresponding to Figure 5 on a laptop equipped with a Intel Core i7-6820HK CPU@2.70 GHz. This was obtained with the image sequence martin-walk (200 × 200 pixels, 41 frames) and the cat_X_cell_DLIF.xml retina configuration file (100 neurons, sparse connectivity with 1,000 connections out of 10,000 possible connections).

## 4. Conclusion

In this paper we have introduced PRANAS, a new free^10^ platform for retinal analysis and simulation. PRANAS provides a retina simulator allowing to transform any video into spike trains, together with sophisticated methods to analyse spike trains, coming from simulations but also experiments. As explained, PRANAS (version 1.0.0) includes a range of original methods from the state-of-the-art (Wohrer and Kornprobst, 2009; Cessac and Palacios, 2012) which are now available to the neuroscience community. More precisely, PRANAS intends to be useful for the neuroscience community in a large sense, i.e., not only experienced computational neuroscientists. To do so, we decided to make all methods implemented therein accessible through a friendly GUI. No scripting is needed to run analysis and simulation methods. This represents a major advantage to promote interdisciplinary neuroscience and we believe that PRANAS helps in this direction.

However, we are certainly aware that this advantage could also be seen as a limitation of the software. Indeed, inability to script analyses could be a limitation that can make systematic analysis of large datasets tedious and error prone. It makes also difficult to link the functionalities of this toolbox to more extensive data analysis workflow. For thoses reasons, we also offer some functionalities that run on the command line. A non-exhaustive list of tools that can be used from the command line is SimulateRetina (simulate retinal response), Correlations (compute the average cross-correlations of a given raster), SpikeTriggerAverage (compute the average spike triggered stimuli)^11^. We expect to have the platform fully available with scripting in a forthcoming version.

In this first release of PRANAS, we have focused on the following aspects:

- The user experience: We optimized the ergonomics of interactions between the user and the interface. No scripting is needed to analyse or generate the spike trains.
- The richness of toolboxes: We developed a variety of methods ranging from classical tools to population analysis, including a method for receptive fields estimation. In addition, we provide two simulators, one inspired in the retina, the other from rasters statistics analysis.
- The computational performance: We parallelized a selection of functions to make possible to handle faster large populations of neurons^12^.

By putting together functions related to retina simulation and analysis, PRANAS intends to be an original platform that will encourage joint modeling and experimental studies. The synergy between these two areas of functionality will be in particular useful to define better retina simulators by confronting simulated output w.r.t. real cell recordings. A typical use case is to start from a real cell recording of a retina, to analyse its receptive field structure, and use that information to define the XML retina configuration file so to define a virtual retina having similar characteristics as the real one. Another use case that we target is to start again from a real cell recording of a retina, analyse spike train statistics and compare them with the ones given by the simulator. Such a comparison is very informative and will suggest improvements of the retina simulator to increase its biological plausibility.

Here, we presented its main features, and we refer the interested reader to its website^13^ for downloads and more information (see e.g., tutorials to get started). We hope that PRANAS becomes a useful tool for neuroscientists to analyse spike trains and we expect to improve it thanks to the users' feedback. Our goal is to progressively enrich PRANAS with the latest research results, to facilitate the transfer of new methods to the community. Future work will focus on improving several existing functions regarding efficiency in time and memory consumptions. We will include methods for population analysis in the non-stationary case as well as other statistical analysis models. We are working on better methods for receptive fields estimation (Drogoul et al., 2016). We are also developing extensions of the retina simulator model so that it could serve as a satisfactory model at a population level. Finally, as a general objective, we also plan to improve the computational power of PRANAS in order to handle even larger networks.

## Author contributions

BC supervised the general development of PRANAS and has especially worked on the population analysis toolbox. PK contributed to the GUI design and is one of the developers of VIRTUAL RETINA. SK contributed to the software development, packaging, and GUI design. HN contributed to the population analysis toolbox. DP contributed to the receptive field and classical analysis toolboxes and to the integration and extension of VIRTUAL RETINA in PRANAS. GP contributed to the GUI design and the classical analysis toolbox. TV contributed to the software development. The authors are listed in alphabetical order.

### Conflict of interest statement

The authors declare that the research was conducted in the absence of any commercial or financial relationships that could be construed as a potential conflict of interest.

## Footnotes

^1^VIRTUAL RETINA website: http://virtualretina.inria.fr

^2^Note that this toolbox first motivated the creation of the GUI to manipulate these complex notions. In former publications, software was named ENAS standing for Event Neural Assembly Simulations. After fusing of ENAS and the retina simulator VIRTUAL RETINA (see Section 3.3.2) into the same platform, we decided to choose a better suited name (PRANAS) to describe the functional scope of this unique platform allowing to (i) analyse spike trains coming from simulations or MEA recordings, at single cells or population levels, (ii) simulate retinal spike trains from a bio-plausible model, (iii) use additional dedicated tools for retinal recordings.

^3^The model initially proposed by Ising and Lenz in 1920 had only nearest neighbors interactions and constant couplings. The potential form we use corresponds in fact to a spin-glass but we use here the terminology found in computational neuroscience papers.

^4^The product *N* × *R* somewhat defines the complexity of the model. The larger *N* × *R* the longer the simulation and the memory load. We have been able to run up to *N* = 100, *R* = 2 in reasonable times. Note however that the statistical estimation of a so-huge number of neurons raises the classical problems (*not inherent to our method*) of statistical estimation on a sample, which, e.g., for the retina rarely exceeds a few millions of spikes.

^5^The Hellinger distance between the empirical probability π and the Gibbs probability μ is $d\left(\pi ,\mu \right)=\frac{1}{\sqrt{2}}\sqrt{\overset{L}{\underset{l=1}{\sum }}{\left(\sqrt{\pi \left(m_{l}\right)}-\sqrt{\mu \left(m_{l}\right)}\right)}^{2}}$.

^6^To test this example, one can use sample data available on PRANAS website: recording called mouse-P39_17_06_14 (We are indebted to Evelyne Sernagor and Gerrit Hilgen, Newcastle University, who provided us these data and authorized us to use them as a basis for examples.)

^7^VIRTUAL RETINA website: http://virtualretina.inria.fr. VIRTUAL RETINA is under Inria CeCILL-C license, IDDN.FR.001.210034.000.S.P.2007.000.31235.

^8^To run this example one can use data available on PRANAS website, for example the martin-walk image sequence and the cat_X_cell_DLIF.xml retina configuration file which corresponds to the result shown in Figure 5.

^9^Note that steps 4–6 can be skipped and one can directly generate spikes in step 7 using a proposed default configuration.

^10^PRANAS is under Inria CeCILL-C license, IDDN.FR.OO1.190004.000.S.P.2014.000.31235. Binaries can be freely downloaded.

^11^These commands can be found in the directory pranas/bin. Help of each tool is given with the command line: toolName -h.

^12^Parallelization concerns every loop in our C++ code where iterations can be computed independently. To do so, we used OpenMP so that PRANAS parallel processes can run on a personal computer equipped with multi-core processors. More precisely, four functions were parallelized: (i) the loading of rasters for all file formats, (ii) the statistical analysis functions to generate potentials, compute divergence and estimate patterns probabilities, (iii) the estimation of receptive fields and (iv) the retina simulator.

^13^PRANAS website: http://pranas.inria.fr
